# Harsh nutritional environment has positive and negative consequences for family living in a burying beetle

**DOI:** 10.1002/ece3.9699

**Published:** 2023-01-06

**Authors:** Eva M. Keppner, Melina Laubenthal, Madlen A. Prang, Taina Conrad, Sandra Steiger

**Affiliations:** ^1^ Institute of Evolutionary Ecology and Conservation Genomics Ulm University Ulm Germany; ^2^ Department of Evolutionary Animal Ecology University of Bayreuth Bayreuth Germany

**Keywords:** biparental care, competition, cooperation, family life, *Nicrophorus*, resource availability

## Abstract

Harsh environmental conditions in form of low food availability for both offspring and parents alike can affect breeding behavior and success. There has been evidence that food scarce environments can induce competition between family members, and this might be intensified when parents are caring as a pair and not alone. On the other hand, it is possible that a harsh, food‐poor environment could also promote cooperative behaviors within a family, leading, for example, to a higher breeding success of pairs than of single parents. We studied the influence of a harsh nutritional environment on the fitness outcome of family living in the burying beetle *Nicrophorus vespilloides*. These beetles use vertebrate carcasses for reproduction. We manipulated food availability on two levels: before and during breeding. We then compared the effect of these manipulations in broods with either single females or biparentally breeding males and females. We show that pairs of beetles that experienced a food‐poor environment before breeding consumed a higher quantity of the carcass than well‐fed pairs or single females. Nevertheless, they were more successful in raising a brood with higher larval survival compared to pairs that did not experience a food shortage before breeding. We also show that food availability during breeding and social condition had independent effects on the mass of the broods raised, with lighter broods in biparental families than in uniparental ones and on smaller carcasses. Our study thus indicates that a harsh nutritional environment can increase both cooperative as well as competitive interactions between family members. Moreover, our results suggest that it can either hamper or drive the formation of a family because parents choose to restrain reproductive investment in a current brood or are encouraged to breed in a food‐poor environment, depending on former experiences and their own nutritional status.

## Graphical abstract

In this study, we showed that a harsh nutritional environment can have different effects on the breeding success of biparental pairs or single females. Burying beetle pairs that experience nutritional stress before breeding rear larvae with a higher survival rate compared to larvae of well‐fed pairs. Additionally, we showed that competition between family members is intensified on small carcasses and females raise bigger broods than pairs.

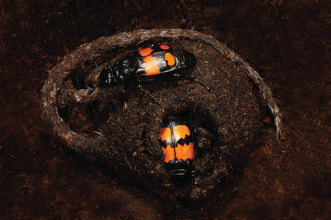

## 1 INTRODUCTION

The evolution of family life and especially (bi‐)parental care in various taxa of insects has received rising interest in the past years. Four “prime movers” are often stated as the drivers of this evolution: stable and structured environments, harsh environmental conditions, specialized food sources, and a high predation risk to eggs (Wilson, 1975). The second mentioned driver, harsh environmental conditions, is usually described as some kind of unfavorable abiotic condition, like heat stress or the risk of desiccation (Wong et al., 2013). Shield bug mothers that relocate their nest sites if physical conditions worsen for the offspring is one of many examples of caring behaviors that strongly supports the role of these abiotic factors (Filippi‐Tsukamoto et al., 1995). However, harsh environmental conditions do not necessarily have to be abiotic. The availability of food within the environment for parents and offspring might be an important factor as well (Wilson, 1975; Wong et al., 2013). This holds true, of course, when parental attendance and care is necessary to locate scarce or scattered resources and these resources have to be made available and usable to the offspring (Tallamy & Wood, 1986). However, feeding is (often) also necessary, if not essential, for the adult individuals themselves and a low food availability due to a resource‐poor environment could lead to a lower somatic condition. Parental care is usually thought to be energetically costly and can also lead to a reduced somatic condition of parents (Alonso‐Alvarez & Velando, 2012). Thus, if adequate food is scarce and the individuals are not optimally nourished, there are several possible scenarios. Family life, the association of one or both caring parent(s) with their offspring after hatching or birth, could be favored, because parental care is directed toward the offspring (i.e., cooperation between parents and their offspring; Kramer & Meunier, 2019) and cooperation between siblings could buffer against these harsh nutritional conditions by, e.g., a more efficient exploitation of resources or cooperative foraging activities (Falk et al., 2014; Kuitunen et al., 1996; Marti, 1989). Additionally, not only family living per se could be promoted by a food‐poor environment, but it could select for biparental care over uniparental care. Two parents are likely more efficient in finding or processing food. Furthermore, parents could also compensate for each other's condition if one parent is too weak to perform all necessary care behaviors alone (Barta et al., 2014; Creighton et al., 2015; Houston et al., 2005; Pilakouta et al., 2018; Smiseth et al., 2005). However, recent studies showed that family living can sometimes lead to competition over food between family members (Keppner et al., 2018; Kramer et al., 2017). Therefore, another possible outcome is that harsh nutritional conditions can also hamper the emergence of family life or favor uni‐ over biparental care, because competition between the family members should increase with the number of parents (Gray et al., 2018).

**FIGURE 1:**
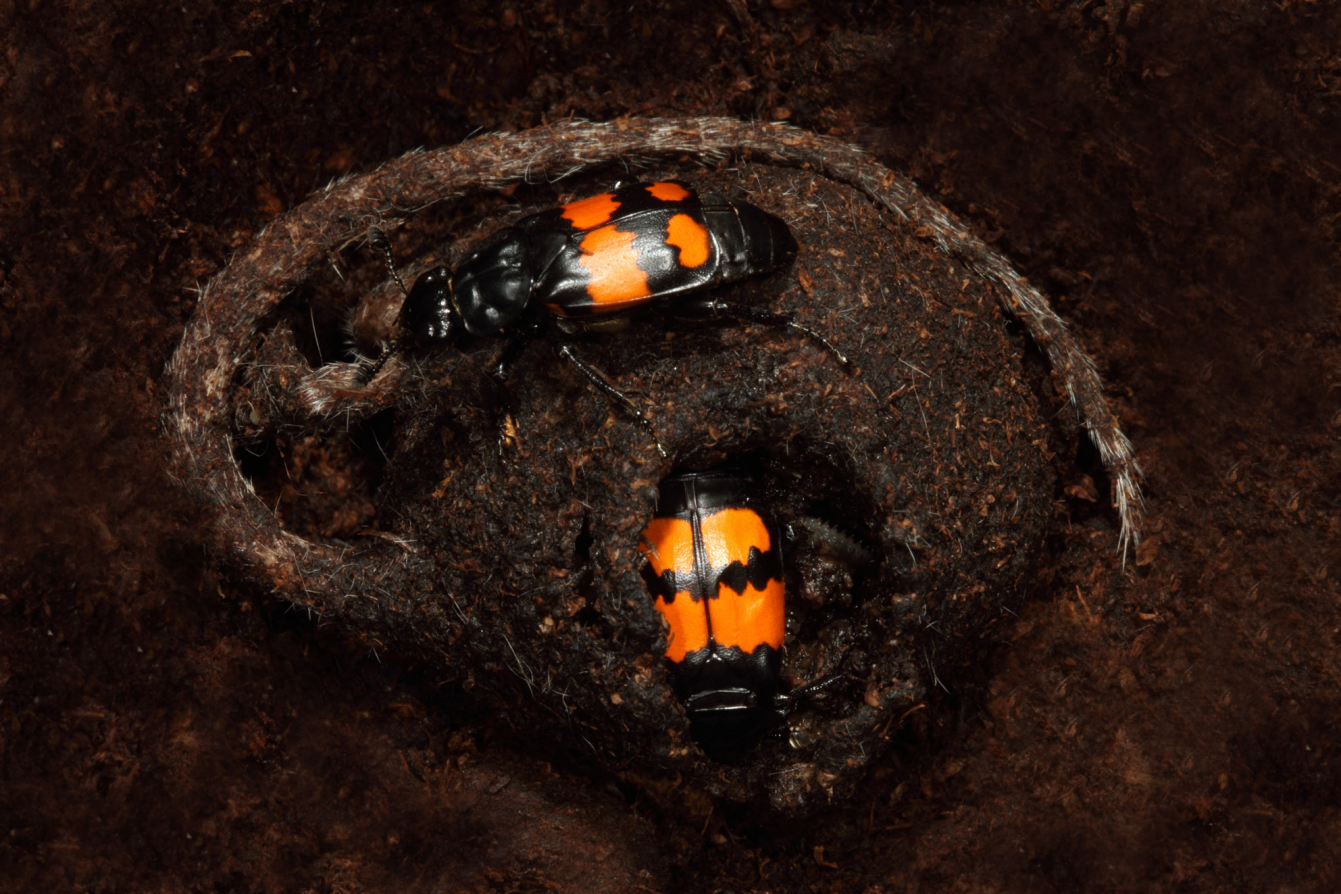
A male and female pair of *Nicrophorus vespilloides* preparing and maintaining a mouse carcass during post‐hatching care (photo by Heiko Bellmann).

Whether a resource‐poor environment leads to more cooperation or competition between family members and whether it promotes or impedes family life and especially biparental care is still an open question. To address this gap in knowledge we used the burying beetle *Nicrophorus vespilloides* as a model system (Figure 1). *N. vespilloides* beetles perform elaborate, facultative bi‐ or uniparental care using small vertebrate carcasses and are able to breed multiple times per season (Eggert & Müller, 1997; Pukowski, 1933). Once a single female or a male–female pair locates a suitable carcass, they will start to bury and prepare it and the female commences egg laying in the surrounding soil (Pukowski, 1933). The decision to start egg laying and the size of the clutch seems to be dependent on the size of the carcass, and very small carcasses are sometimes rejected and used for feeding and not for reproduction (Müller et al., 1990). Both the male and female parent can adjust the number of larvae to the available amount of carcass and will perform infanticide after larval hatching if the number of larvae is too high (Bartlett, 1987; Müller et al., 1990). The parents feed from the carcass and provide the larvae with regurgitated food, but larvae are also able to self‐feed. During breeding, all family members solely rely on the carcass as food source and do not forage away from the brood (Eggert & Müller, 1997; Scott, 1998), which makes them an ideal organism to study questions related to the relationship of food availability and family life. There is already some evidence for competition for food from the breeding resource between the parents and between the parents and the offspring (Keppner et al., 2018; Pilakouta et al., 2016). However, it is unclear whether such competition due to food limitation might prevent the formation of a family or, if a family is started despite the competition, might promote uniparental care compared to biparental. There is also some evidence for synergistic effects when parents raise offspring together (Pilakouta et al., 2018), but whether cooperation can buffer against decreased care quality due to poor nutritional condition and therefore favors biparental over uniparental care is unclear.

To address the question whether harsh nutritional conditions lead to a higher degree of cooperation or competition among family members, we manipulated food availability on two levels – before breeding, in terms of the food supply for adult beetles, and during breeding, in terms of carcass size and therefore food availability for offspring and parents alike. Additionally, we manipulated family social condition, letting the beetles either care bi‐ or uniparentally. If synergistic effects among family members play a major role, we would expect that under poor nutritional condition pairs raise a higher number of larvae and/or broods with an overall lower mass compared to single beetles. This effect should vanish or be less pronounced on larger carcasses, as parents can compensate for their poor nutritional condition by feeding from the carcass without inflicting costs to the offspring. If food competition among family members plays a major role, we would expect that pairs are more likely to have a lower brood success under poor nutritional conditions than single beetles and that this effect would be more pronounced on smaller carcasses. At larger carcasses, where food is more abundant, we would expect to see a smaller or no effect of family social condition or food availability prior to breeding on brood success. To test our “competition hypothesis” we also measured how much food from the carrion resource was removed to see whether pairs consume more than single beetles and whether the pre‐breeding nutritional condition of the prospective parents plays a role.

## 2 MATERIAL AND METHODS

### 2.1 Origin and maintenance of experimental beetles

Experimental *N. vespilloides* were descendants (5th generation) of wild beetles collected from carrion‐baited pitfall traps in a forest near Bayreuth, Germany (“Studentenwald”; 40°55′19″N, 11°34′21″E). We avoided inbreeding by haphazardly pairing beetles which were neither cousins nor siblings. All beetles were maintained in temperature‐controlled chambers at 20°C under a 16:8 h light: dark cycle in plastic containers (10.0 × 10.0 × 6.5 cm) filled with moist peat and were fed with cut darkling beetle larvae (*Zophobas morio*) twice a week prior to the start of manipulations. All beetles used in this study were 20–40 days old and had never reproduced prior to the experimental procedures. The experiments were conducted from May until August 2020. Only one temperature‐controlled chamber was used for the experiment and breeding boxes were randomly distributed within the chamber.

### 2.2 Experimental design

**FIGURE 2:**
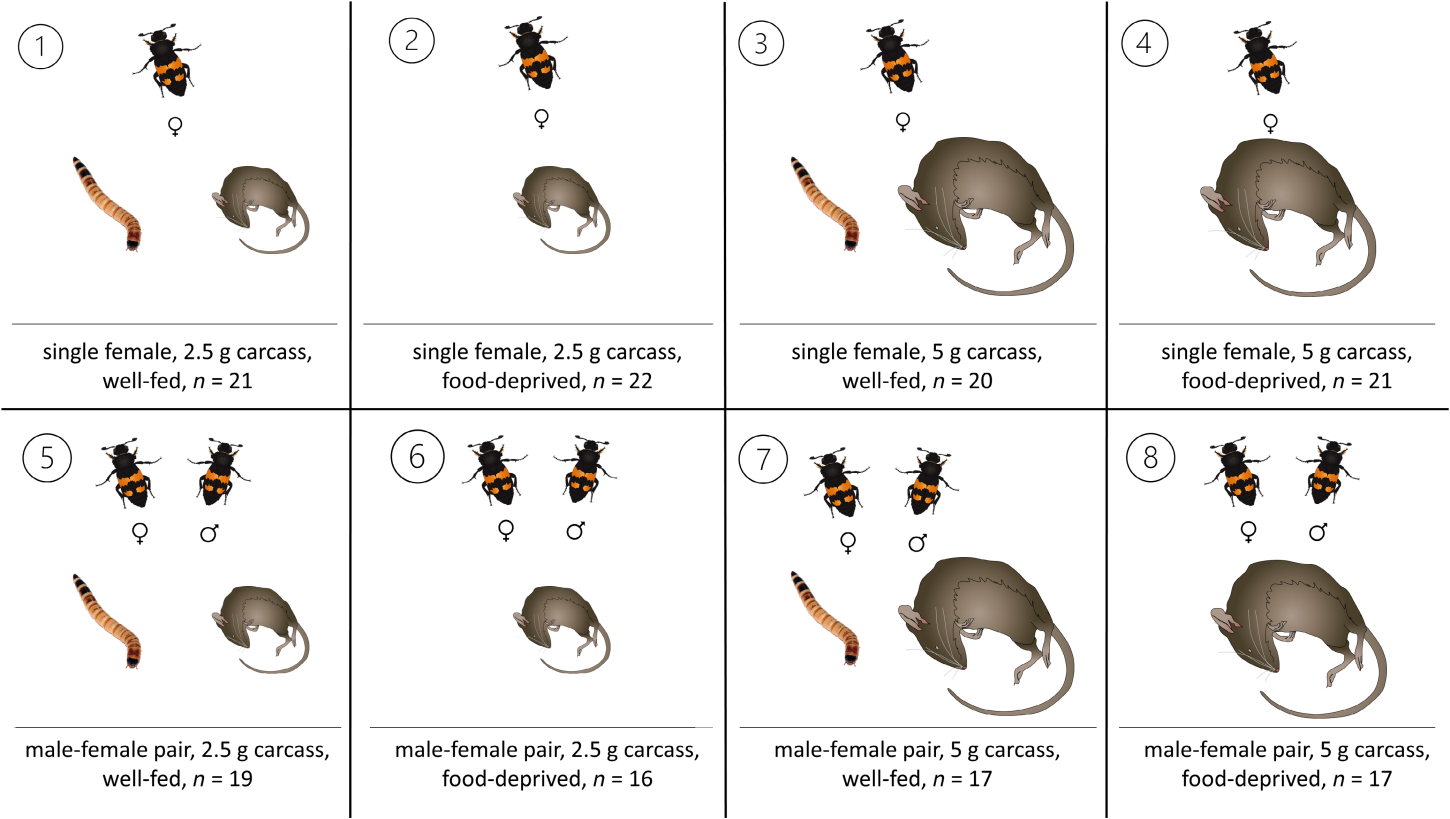
Schematic illustration of our experimental groups. Beetles were exposed to eight different environments that offer different scope for competition or cooperation over the resources. We manipulated the nutritional condition of the parents before breeding (well‐fed vs. food‐deprived), the food availability during breeding (carcass size 2.5 g vs. 5 g), and the social condition (biparental pair vs. uniparental female). Sample size is included as *n*. Sample size indicates group sample size *after* removing broods due to the death of parent beetles.

To test whether a harsh, resource‐poor environment hampers or promotes family living and biparental care by inducing a higher degree of either cooperative or competitive interactions between the family members, we designed a laboratory experiment where we manipulated the nutritional condition of the parents before breeding (well‐fed vs. food‐deprived), the food availability during breeding (carcass size of 2.5 g vs. 5 g) and the social condition (biparental pair vs uniparental female). To start the experiment, we placed pairs of beetles in boxes (10 × 10 × 6 cm) filled with moist peat. We paired males and females for three days to ensure that females receive enough sperm to fertilize their eggs. During these three days, half of the beetles were fed with a diet of darkling beetle larvae (*Zophobas morio*) ad libitum to generate well‐fed beetles and the other half did not receive any food to generate food‐deprived beetles. After those three days the beetles were separated to prevent lethal fights between the pairs and cannibalism. The beetles remained in their nutritional status group for additional four days before single females or male–female pairs were put in a new box and provided with either a 2.5 g (2.54 ± 0.21 g) or a 5 g (5.43 ± 0.31 g) mouse carcass. In total, the food‐deprived group did not receive food for seven days. This amount of time is sufficient to lead to a weight loss, but is usually not lethal (Chemnitz et al., 2015; Steiger et al., 2007). We weighed the beetles before and after these seven days. The two carcass sizes we chose for our experiment fall at the lower end of the preferred size range of carcasses which *N. vespilloides* chooses for reproduction in the wild (Müller et al., 1990). We chose these sizes to create an extreme (2.5 g) or moderate (5 g) food scarcity for the brood. Using this set up, we created 8 different treatment groups (Figure 2). The size of beetles (pronotum width) did not differ between the experimental groups (one‐way ANOVA: female size: *F*_7,145_ = 1.49, *p* = .176; male size: *F*_3,63_ = 0.69, *p* = .56). We removed all broods from the analysis, where the parent (or at least one parent in the biparental group) died during the experiment (9.6% of broods where parents died before receiving the carcass and 9.0% of broods where parents died after receiving the carcass) and ended up with a total of 153 broods (see Figure 1 for details on groups and sample sizes). Whether a beetle died before or after carcass receival was not influenced by nutritional status before breeding (Chi^2^‐Test: ${Χ}_{1;35}^{2}$ = 1.37; *p* = .24) or social condition (Chi^2^‐Test: ${Χ}_{1;35}^{2}$ = 0.014; *p* = .91); note that in broods with biparentally caring parents the probability of death of at least one partner was twice as high as for single beetles. To take this difference of mortality probability into account, we multiplied the number of uniparental deaths by two. Therefore, we assume that beetle deaths occurred to reasons not connected to our experimental treatments. We monitored all groups the same way under dim red light to keep disturbance at a minimum. After 48 h, we transferred the beetles along with the carcass to a new, fresh container, also filled with moist peat. We measured the weight of the carcass, the weight of the beetles and counted the eggs we found in the old container and determined clutch size. After counting, we placed the eggs in the soil surrounding the carcass in the new container. *N. vespilloides* females usually lay their eggs asynchronously within the first 48 h. However, to ensure that we did not underestimate clutch size in case some females had started egg laying later, we repeated our procedure again after an additional 48 hours if clutch size was below 10. If clutch size was still below 10, we repeated the procedure 24 h later. Pairs or single females without any eggs by this time were not further observed. We left the remaining broods undisturbed until the larvae started to disperse from the carcass. To measure brood success, we counted and weighed the larvae at the time of larval dispersion. We used a Kern ABJ‐NM precision scale to measure weight of beetles (to the nearest 0.0001 g), larvae (to the nearest 0.0001 g) and carcasses (to the nearest 0.001 g) and Profiscale Precise PS 7215 digital calipers to measure pronotum width of beetles (to the nearest 0.01 mm).

### 2.3 Statistical analyses

All analyses were conducted using R version 4.0.3 (R Core Team, 2020). First, to check whether the manipulation of the pre‐breeding nutritional condition was successful in terms of a decline in body mass, we performed a GLM with Gaussian error structure and a logit link function with the change in body mass as response variable and the nutritional condition (food‐deprived or well‐fed), sex and body size (pronotum width) as categorical variables.

Next, we tested if our manipulations influenced the amount of carcass mass that was consumed by the adult beetles of the different groups in the first 48 hours. We conducted a GLM with Gaussian error structure with the decline in carcass mass as response variable and carcass size, nutritional condition, and social condition (uni‐ or biparental) as explanatory variables.

To test whether our stimulated harsh environments have positive or negative effects on family life, we tested for effect on clutch size, larval survival rate, and brood mass. We first conducted a GLM with clutch size (number of eggs laid) as response variable, and social condition, carcass size, and nutritional condition as explanatory variables using a quasi‐Poisson error structure (to account for count data and overdispersion of the parameter) and a logit link function. We additionally tested whether there was a difference between the experimental groups with regard to pairs or females with zero eggs. We therefore performed a GLM with the binary response variable eggs (eggs present = 1; no eggs = 0) and social condition, carcass size, and nutritional condition as explanatory variables using a quasibinomial error structure (to account for binary data) and a logit link function. Continuing from here, we tested whether our stimulated harsh environment influenced the survival rate of the larvae. This response variable was calculated as the number of larvae that survived until dispersal in relation to the number of eggs initially laid. Hence, this variable controlled for any differences in fecundity among females. Pairs or females without eggs were not included in the analysis. We conducted a GLM with proportion of surviving larvae as response variable and social condition, carcass size, and nutritional condition before breeding as explanatory variables using a quasibinomial error structure (to account for proportion data) and a logit link function. Finally, we conducted a GLM with total brood mass as response variable and social condition, carcass size, and nutritional condition as explanatory variables using a Gaussian error structure and a logit link function. Pairs or single females that did not produce any eggs or larvae were not included in the analysis.

The statistical significance of models without interaction terms was obtained from type II sums of squares, whereas significance for models with interaction terms was obtained from type III sums of squares, all by using the R package *car* (Fox et al., 2012). We first tested all GLMs including all possible interaction terms. If no interactive effects were present, we removed the interaction terms from the model. We furthermore performed post hoc tests on statistically significant variables to compare the differences between the groups using the *emmeans* command of the R package emmeans (Lenth, 2022). Holm method for *p*‐value adjustment to correct for multiple comparisons was used.

## 3 RESULTS

### 3.1 Effects of a harsh nutritional environment on adult body mass and carrion consumption

The manipulation of the pre‐breeding nutritional environment significantly affected the body mass of the parents, with an overall decrease in body mass in the food‐deprived beetles compared to an increase in body mass in the well‐fed beetles (*F*_1,218_ = 166.44, *p* < .0001; body mass change of food‐deprived beetles: mean ± SD = −30.9 ± 12.4 mg; body mass change of well‐fed beetles: 9.2 ± 29.9 mg; Figure S1). Body size (*F*_1,218_ = 2.2, *p* = .1) or sex of the beetle (*F*_1,218_ = 2.8, *p* = .14) had no effects on body mass change. Therefore, we assume that the manipulation was successful and led to a decline in nutritional state of the beetles in the food‐poor pre‐breeding environment.

**FIGURE 3:**
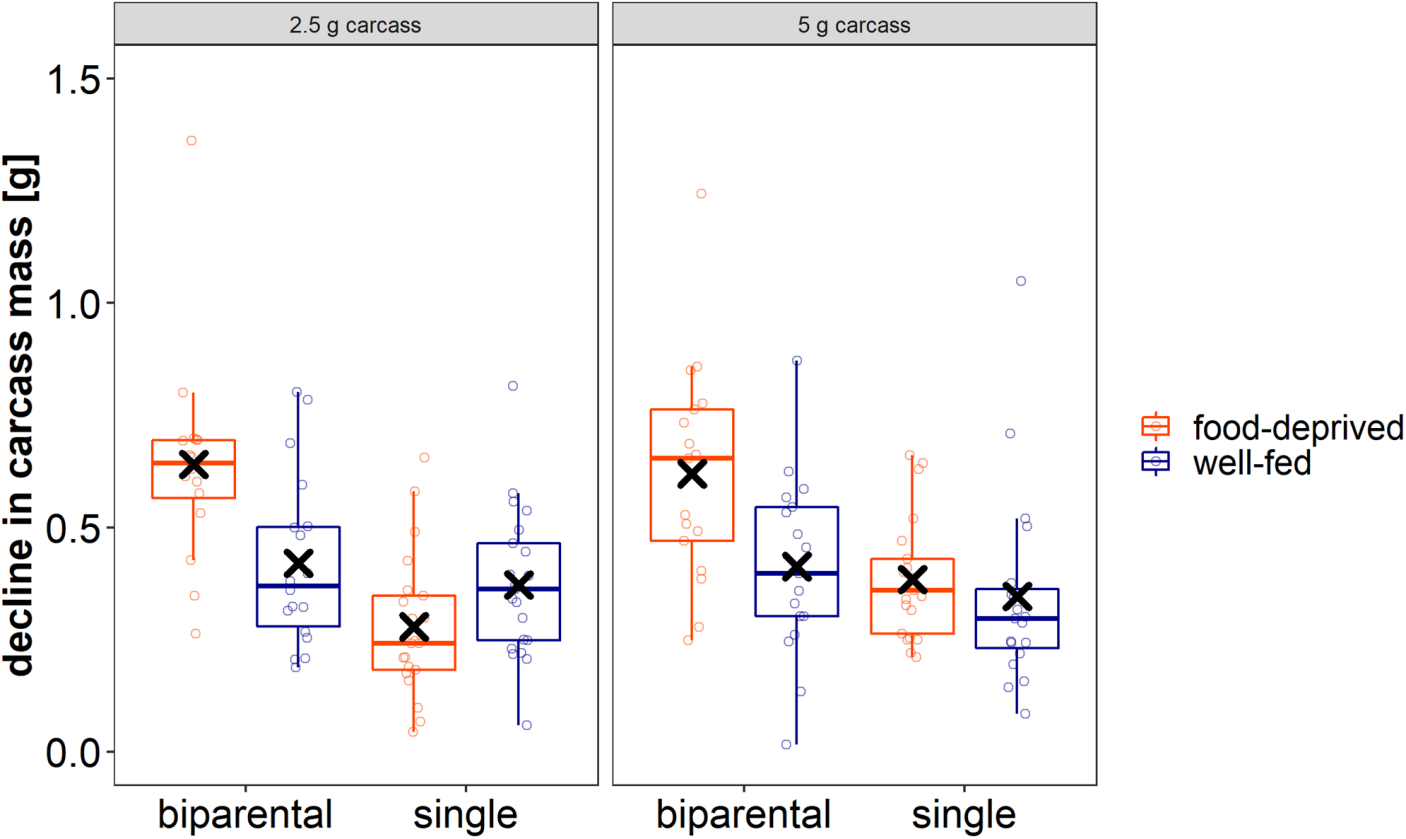
The impact of social and nutritional condition on the decline in carcass mass (g) in the first 48 h after the start of carcass preparation. The left panel shows the decline in carcass mass on 2.5 g carcasses, the right on 5 g carcasses. Boxplots show median, interquartile range, minimum/maximum range. Points indicate the original data points. Mean values are indicated by x.

The amount of carrion consumption until larval hatching significantly differed between the experimental groups (Table 1; Figure 3). There was no effect of carcass size on the amount of carrion consumed. We found, however, a significant effect of the interaction between the nutritional condition before breeding and the social condition on the amount of carrion consumed (Table 1; Figure 3). Beetles caring biparentally consumed more food than uniparental females but only when they had been food deprived. In fact, we found that biparentally caring, food‐deprived beetles consumed more than all the other groups (Figure 3; post hoc test: Table S1; Figure S2).

**TABLE 1:** Effect of manipulations of nutritional and social environment on carcass consumption

Response variable		Explanatory variable							Model adjusted *R* ^2^
		Nutritional condition	Social condition	Carcass size	Nutritional condition × social condition	Nutritional condition × carcass size	Social condition × carcass size	Nutritional condition × social condition × carcass size	
Decline in carcass mass (*n* = 144)	*df*	1	1	1	1	1	1	1	
	*F*	12.16	30.87	0.09	14.17	0.14	2.22	2.35	0.23
	*p*	**<.001**	**<.0001**	.762	**<.001**	.709	.139	.127	

*Note*: Results of the GLMs of the effect of social condition (uni‐ vs. biparental care), nutritional condition (food‐deprived vs. well‐fed) and carcass size (2.5 and 5 g) and the interaction of these parameters on the decline in carcass mass. Significant *p*‐values are typed in bold.

### 3.2 Effects of a harsh nutritional environment and social condition on clutch size and larval survival rate

**FIGURE 4:**
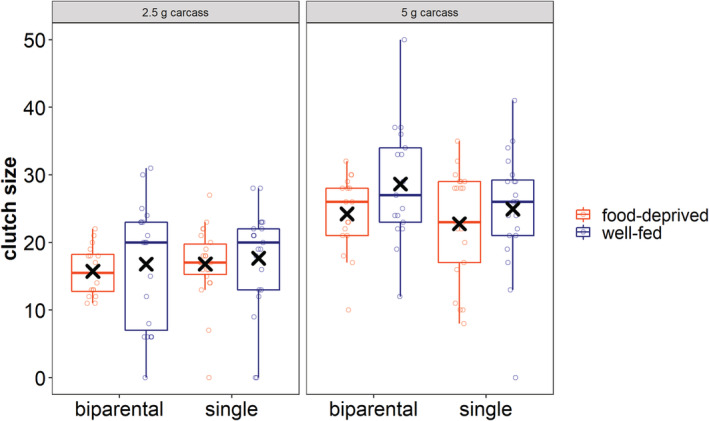
The impact of social and nutritional condition on clutch size. The left panel shows clutch sizes produced on 2.5 g carcasses, the right on 5 g carcasses. Boxplots show median, interquartile range, minimum/maximum range. Points indicate the original data points. Mean values are indicated by x.

Clutch size was highly influenced by carcass size with more eggs in clutches on 5 g carcasses than on 2.5 g carcasses (Table 2; Figure 4; post hoc test: Table S2, Figure S3). Whether the beetles were well‐fed or food‐deprived before breeding or whether they cared as pairs or not had no influence on the number of eggs laid. Only 5 out of 153 females did not produce any eggs. Hence, whether eggs were produced or not did not depend on the nutritional environment before or during breeding or the social condition (Table 2).

**TABLE 2:** Effect of manipulations of nutritional and social environment on breeding performance

Response variable		Explanatory variables							Model adjusted *R* ^2^
		Nutritional condition	Social condition	Carcass size	Nutritional condition × social condition	Nutritional condition × carcass size	Social condition × carcass size	Nutritional condition × social condition × carcass size	
“Eggs” (yes/no; *n* = 144)	*df*	1	1	1					
	*F*	2.79	2.15	2.56					.1
	*p*	.097	.14	.11					
Clutch size (*n* = 144)	*df*	1	1	1					
	*F*	2.58	0.48	46.07					.18
	*p*	.11	.49	**<.0001**					
Larval survival rate (*n* = 139)	*df*	1	1	1	1	1	1	1	
	*F*	8.2	0.65	16.25	6.11	1.37	0.47	3.15	.27
	*p*	**.005**	.42	**<.0001**	**.015**	.25	.49	.07	
Brood mass (*n* = 113)	*df*	1	1	1					
	*F*	1.68	8.57	223.28					.65
	*p*	.20	**.0041**	**<.0001**					

*Note*: Result of the GLMs of the effect of social condition (uni‐ vs. biparental care), nutritional condition (food‐deprived vs. well‐fed) and carcass size (2.5 g vs. 5 g) and the interaction of these parameters (only if significant) on the binary parameter “eggs”, clutch size, larval survival rate, and brood mass. Significant *p*‐values are typed in bold.

**FIGURE 5:**
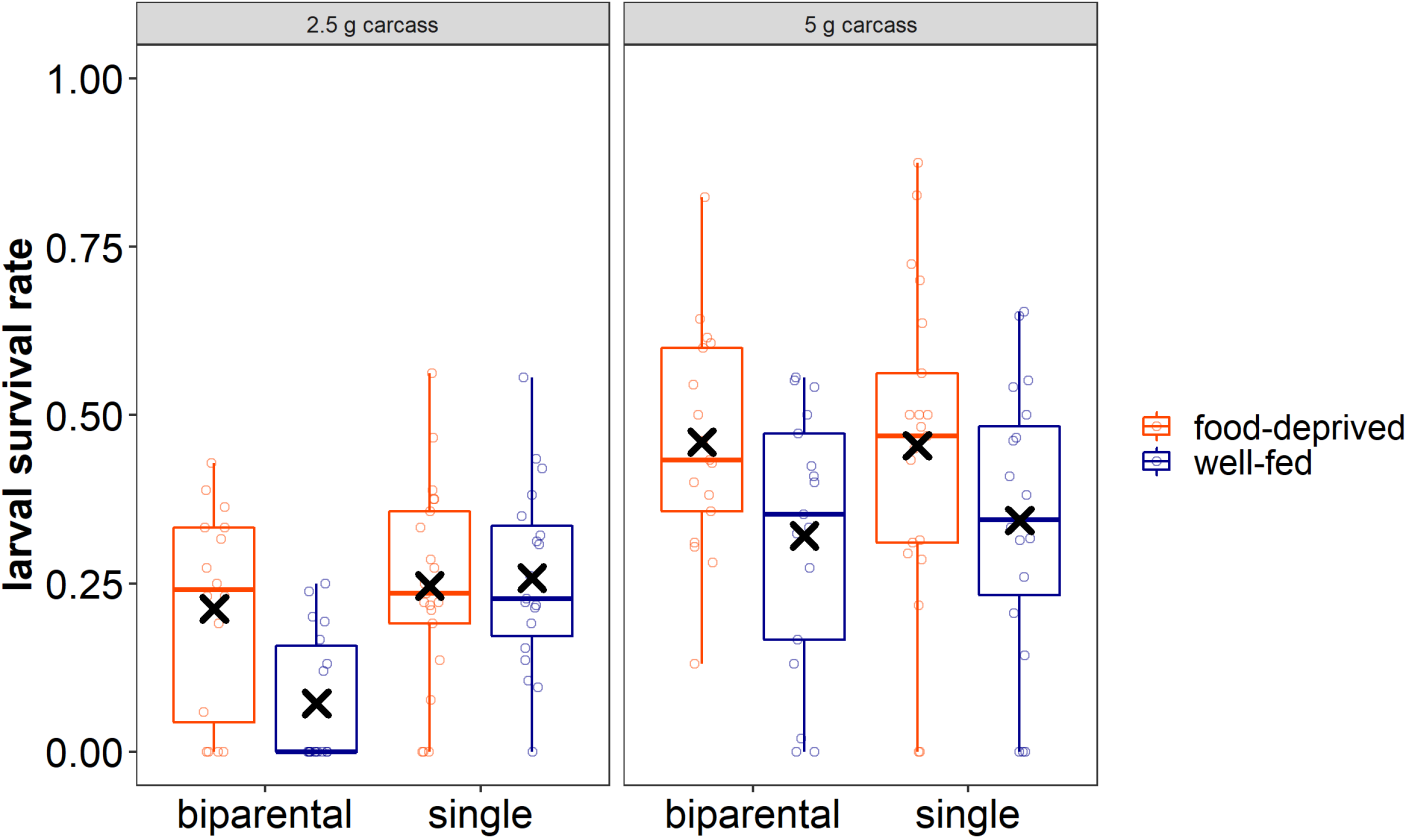
The impact of social and nutritional condition on larval survival rate. The left panel shows the larval survival rate on 2.5 g carcasses, the right on 5 g carcasses. Boxplots show median, interquartile range, and minimum/maximum range. Points indicate the original data points. Mean values are indicated by x.

Our results show that larval survival rate until dispersal differed between the experimental groups. Carcass size significantly influenced the larval survival rate, with more larvae surviving on larger carcasses (Table 2; post hoc test: Table S3.1; Figure S4.1). We also found a significant effect of the nutrition before breeding and of the interaction between the social condition and the nutrition before breeding on the larval survival rate (Table 2; Figure 5). The significant interaction reflected that larvae from biparentally caring parents had a lower survival rate than those from single females, but only when the parents had been well nourished before breeding. In fact, well‐fed, biparentally caring beetles raised larvae with a significantly lower survival rate than all the other groups, including food‐deprived pairs, even though the latter group consumed more food from the carcass resource (post hoc test: Table S3.2; Figure S4.2).

### 3.3 Effects of a harsh nutritional environment and social condition on brood mass

**FIGURE 6:**
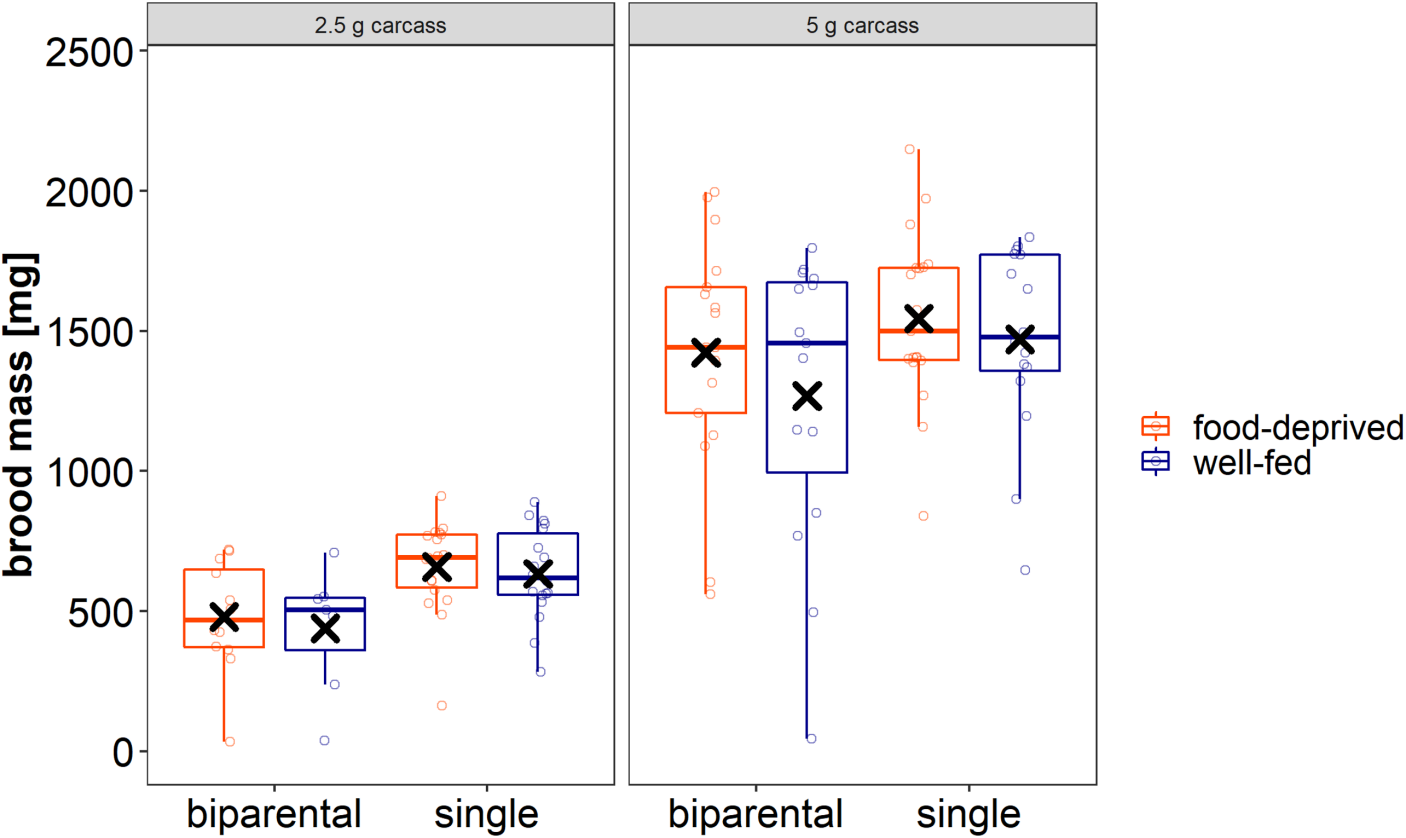
The impact of social and nutritional condition on total brood mass. The left panel shows the total brood mass produced on 2.5 g carcasses, the right on 5 g. Boxplots show median, interquartile range, minimum/maximum range. Points indicate the original data points. Mean values are indicated by x.

Our results showed significant effects of both carcass size and social condition on the parameter brood mass (Figure 6; Table 2). Brood mass was higher on larger carcasses and single females reared larger broods compared to biparental parents (post hoc test: Table S4; Figure S5). Nutritional condition did not affect the total mass of a brood (Figure 6; Table 2).

## 4 DISCUSSION

In this study, we wanted to find out whether a harsh nutritional environment leads to a higher degree of cooperative or competitive effects among family members and thus can either drive or impede the emergence of family life and especially biparental care. We first show that food‐deprived pairs of beetles consumed more from the carcass which they prepare for reproduction than well‐fed pairs or single females. Then, we show that clutch size was not influenced by harsh nutritional conditions before breeding, but by the food availability during the reproductive event, with smaller clutches on smaller carrion resources. As a third result, we demonstrate that well‐fed pairs had the lowest rate of larval survival compared to the other groups, and consequently food‐deprived pairs performed better raising the brood than well‐fed ones. We also show that harsh nutritional conditions during breeding had a negative effect on larval survival rate. Furthermore, we show that, if we only consider the successful broods (i.e., broods with at least one larva surviving until dispersal), single females reared broods with a higher mass than biparentally caring parents. Our study thus does give various indications that harsh environmental conditions can lead to competition between family members: between the parents and between parents and offspring, as pairs of beetles consumed more from the carcass and raised a brood of lower mass than single females, and likely between siblings, as larval survival rate was affected by the size of the carrion resource. However, our study also gives some evidence that cooperation is favored under certain circumstances of a harsh environment, as food‐deprived pairs of beetles have a higher rate of larval survival than well‐fed pairs. Below we provide a more detailed discussion of our results and their wider implications for our understanding of the emergence of family life.

Before the larvae hatch, burying beetles prepare the carcass and feed from it at the same time (Trumbo & Xhihani, 2015). They probably use this timeframe to try to replenish their energy reserves to be able to successfully breed. Our first finding showed that the food availability prior to breeding can influence the rate of self‐feeding at the carcass, but only if the beetles are caring biparentally. More precisely, we found that food‐deprived pairs consumed more than the other experimental groups during the period of pre‐hatching care. Hence, on the one hand, food‐deprived parents might benefit from the help of a second parent as parental tasks can be divided or shared, but on the other hand, a second parent also feeds from the limited resource and could lead to increased competition among family members (Keppner et al., 2020). Interestingly, there was no difference between well‐fed females and food‐deprived females in the amount of carcass consumed. This might be explained by the fact that female burying beetles restrict their own consumption under certain circumstances for the benefit of their offspring (Keppner et al., 2018). In general, our results show that food consumption of the parents during pre‐hatching care is variable and dependent on different factors. This needs to be considered, as it changes the food availability for the larvae during post‐hatching care and might also affect decisions regarding the formation of a bi‐ or uniparental family.

Our second result showed that almost all females start to lay at least one egg irrespective of resource availability before or during breeding or whether they had a male partner when receiving the carcass. Burying beetles are so‐called capital breeders and a certain level of fat stores is necessary for such species to initiate egg laying (Alonso‐Alvarez & Velando, 2012; Richardson et al., 2020). The carcass resource is stringently required for breeding and the carrion meal is essential for the ovarian maturation of female *N. vespilloides* (Wilson & Knollenberg, 1984). In our experiment, the females' conditions seem to be sufficiently good and egg laying is initiated even after receiving a very small cadaver. Family life per se only starts after larval hatching (Kramer & Meunier, 2019). However, laying eggs and starting to prepare a carcass (i.e., the engagement in pre‐hatching care) already shows the intention to start a family, which in our case happens even under different nutritional conditions. We additionally showed that the size of a clutch was not affected by the harsh nutritional environment before breeding, which also confirms the findings of a recent study on reproductive behavior of food‐deprived single females (Richardson et al., 2019). However, the nutritional environment during breeding influenced the amount of eggs, and on smaller carcass sizes, smaller clutches were laid. This adjustment of clutch size on carcass size is an important trait of this species (Müller et al., 1990), because it is one mechanism to optimize the reproductive output on a certain amount of the available resource (Eggert & Müller, 1997).

Whereas the pre‐breeding nutritional environment had no effect on clutch size decisions, it affected the decision on how much the beetles invested in reproduction after the phase of egg laying, which is reflected in our third main finding: our results showed that beetles that were well‐fed and breeding in pairs had the lowest rate of larval survival. This result is, at a first glance, unexpected, because vice versa it means, that caring as a food‐deprived pair leads to an improved rate of larval survival. It is possible that this result is due to a higher degree of cooperative behavior between the parents, initiated by the harsh nutritional environment before breeding. Burying beetles parents try to compensate for their partners handicaps (Creighton et al., 2015; Keppner et al., 2018; Suzuki, 2016), which is, in our case, a bad nutritional condition. Additionally, breeding biparentally leads to synergistic effects in burying beetles and food‐deprived pairs could therefore benefit from each other's help (Pilakouta et al., 2018). Another possible reason for our result of a lower larval survival rate in well‐fed pairs is that the environment the beetles experience prior to breeding can influence breeding and care decisions for upcoming breeding opportunities. Beetles that experienced a food‐poor environment prior to breeding might use the opportunity to breed even on a very small carcass, because the resource‐poor environment led to a less selective behavior and made them accept a wider range of carcass sizes, whereas beetles that experienced a food‐rich environment tend to skip this opportunity or invest less, as they might expect to find a larger carcass in future. In fact, it has already been shown that burying beetles do consider experiences, such as competition with conspecifics (Pilakouta et al., 2016), infection with phoretic mites (De Gasperin et al., 2018), population density and resource quality (Billman et al., 2014; Woelber et al., 2018) in decisions concerning future reproductive events. For example, females breeding on a large carcass produced larger broods after a first breeding attempt on a small carcass, compared to females breeding twice in a row on a large carcass (Billman et al., 2014). Another possible mechanism for our finding is that food‐deprived beetles engaged in terminal investment. Individuals at the end of their life span or those who face harming circumstances have been predicted to enhance their investment in reproduction (Williams, 1966), and there is already some evidence that this holds true for burying beetles (Creighton et al., 2009; Farchmin et al., 2020). The food‐poor environment simulated by our study likely induced a bad somatic condition and the beetles therefore might have invested more in the current brood as future reproduction seemed unlikely due to a reduced life expectancy. Food deprivation does induce a terminal investment strategy also in other species, like, e.g., the yellow mealworm beetle (Krams et al., 2015) or a certain moth species (Javoiš & Tammaru, 2004). However, this explanation is not well supported by our results, as well‐fed single females showed a similar high larval survival rate as food‐deprived pairs. Irrespective of the exact mechanism involved, all mentioned possible mechanisms implicate that cooperation between the parents in form of parental cooperation or between parents and their offspring in form of care was increased by food‐deprived pairs relative to well‐fed ones. Our finding thus has implications for our understanding of the evolutionary dynamics of family life in general. Studies of the factors driving the evolution of family life have often focused on the environmental factors that affect the offspring (i.e., high vulnerability to predation) and focused less on preceding factors affecting the parents. Our study shows that, especially in combination with an ephemeral breeding resource, the biotic environment before breeding can influence the decision on how much to invest in family life and therefore, the fitness outcome of family living.

Our last main finding showed that pairs of beetles reared a brood of lower mass than single females irrespective of the nutritional environment they encountered before or during breeding. The reason for this finding is likely a combination of two factors: (1) food‐deprived pairs consumed more from the carcass than single females, with the result that there was less food left for the larvae to feed on and (2) well‐fed pairs invested less in care than single females. Interestingly, this result seems to be in contrast with earlier findings on burying beetles that show that uni‐ and biparental care lead to the same brood success (Bartlett, 1988; Müller et al., 1998; Smiseth et al., 2005). However, we think that this can be explained by the different carcass sizes used. Those previous studies used large carcasses, we, however, wanted to simulate harsh environments and used very small carcass sizes. These small carcass sizes fall at the lower end of the accepted range of sizes, hence competition between the family members over the resource is very likely (Keppner et al., 2018). This increase in competition could eventually lead to a selection of uniparental care over biparental care when resource availability is low during breeding. Indeed, this notion aligns with the results of Ratz et al. (2021) and Kishida and Suzuki (2010) who found that males left earlier on smaller than on larger carcasses.

At least one of the four proposed prime movers of the evolution of family life mentioned in the introduction poses a challenge to almost all insect species. However, only about 1% of them developed forms of family living and parental care and only a very small subset of species engages in biparental care (Costa, 2006). This ambiguity requires work to find out more about the dynamics that lead to the formation of a family, and we hereby tried to shed some light on this question. Combining our results, we can find two main conclusions: A food‐poor pre‐breeding environment might be an additional mover of family life around ephemeral resources, at least it can positively affect offspring survival rate in biparental families. However, when food availability is low during breeding, uniparental care over biparental care seems to be favored, because this might avoid the negative effects of sexual conflict and reduce competition between the parent and offspring over the feeding resource. Consequently, a harsh nutritional environment can induce both cooperative as well as competitive interactions between family members and therefore plays an important role in family conflict dynamics.

## AUTHOR CONTRIBUTIONS

**Eva M. Keppner:** Conceptualization (equal); formal analysis (equal); methodology (equal); project administration (equal); writing – original draft (lead); writing – review and editing (equal). **Melina Laubenthal:** Formal analysis (equal); investigation (lead); visualization (equal); writing – review and editing (equal). **Madlen A. Prang:** Formal analysis (equal); investigation (supporting); methodology (equal); visualization (equal); writing – review and editing (equal). **Taina Conrad:** Formal analysis (supporting); investigation (supporting); project administration (equal); writing – review and editing (equal). **Sandra Steiger:** Conceptualization (lead); methodology (lead); project administration (equal); supervision (equal); writing – review and editing (equal).

## CONFLICT OF INTEREST

The authors declare that there is no conflict of interest regarding the publication of this article.

## Supporting information


**Figure S1.** Weight of males and females before and after the manipulation of food availability before breeding. Males and females were either well‐fed or food‐deprived. Two connected points show individual beetles’ body mass before and after.
**Table S1.** Carcass consumption during pre‐hatching care: Post hoc test on the interaction of nutrition before breeding and social condition
**Figure S2.** The impact of social condition and carcass size on the decline in carcass mass [g] in the first 48 h after the start of carcass preparation. The left panel shows the decline in carcass mass caused by food‐deprived parents, the right by well‐fed parents. Boxplots show median, interquartile range, food‐deprived parents, the right by well‐fed parents. Boxplots show median, interquartile range, Different letters indicate significant differences (*p* < .05).
**Table S2.** Clutch size: Post hoc test on the parameter carcass size
**Figure S3.** The impact of social and nutritional condition on clutch size. The left panel shows clutch sizes produced on 2.5 g carcasses, the right on 5 g carcasses. Boxplots show median, interquartile range, minimum/maximum range. Points indicate the original data points. Mean values are indicated by x. Different letters indicate significant differences (*p* < .05).
**Table S3‐1.** Larval survival rate: post hoc test on carcass size.
**Figure S4.** The impact of social and nutritional condition on larval survival rate. The left panel shows the larval survival rate on 2.5 g carcasses, the right on 5 g carcasses. Boxplots show median, interquartile range, minimum/maximum range. Points indicate the original data points. Mean values are indicated by x. Different letters indicate significant differences (*p* < .05).
**Table S3‐2.** Larval survival rate: Post hoc test on the interaction of nutrition before breeding and social condition.
**Figure S4‐2.** The impact of nutritional condition and carcass size on larval survival rate. The left panel shows the larval survival rate in biparental broods, the right in uniparental broods. Boxplots show median, interquartile range, minimum/maximum range. Points indicate the original data points. Mean values are indicated by x. Different letters indicate significant differences (*p* < .05).
**Table S4.** Brood mass: Post‐hoc tests on the parameters social condition and carcass size
**Figure S5.** The impact of social and nutritional condition on total brood mass. The left panel shows the total brood mass produced on 2.5 g carcasses, the right on 5 g. Boxplots show median, interquartile range, minimum/maximum range. Points indicate the original data points. Mean values are indicated by x. Different letters indicate significant differences (*p* < .05).

## Data Availability

Data associated with this work are available in the Dryad Digital Repository: https://doi.org/10.5061/dryad.1rn8pk0z7.

## References

[ece39699-bib-0001] Alonso‐Alvarez, C. , & Velando, A. (2012). Benefits and costs of parental care. In N. J. Royle , P. T. Smiseth , & M. Kölliker (Eds.), The evolution of parental care (pp. 40–54). Oxford University Press.

[ece39699-bib-0002] Barta, Z. , Székely, T. , Liker, A. , & Harrison, F. (2014). Social role specialization promotes cooperation between parents. The American Naturalist, 183, 747–761. 10.1086/676014 24823819

[ece39699-bib-0003] Bartlett, J. (1987). Filial cannibalism in burying beetles. Behavioral Ecology and Sociobiology, 21, 179–183.

[ece39699-bib-0004] Bartlett, J. (1988). Male mating success and paternal care in *Nicrophorus vespilloides* (coleoptera: Silphidae). Behavioral Ecology and Sociobiology, 23, 297–303.

[ece39699-bib-0005] Billman, E. J. , Creighton, J. C. , & Belk, M. C. (2014). Prior experience affects allocation to current reproduction in a burying beetle. Behavioral Ecology, 25, 813–818.

[ece39699-bib-0006] Chemnitz, J. , Jentschke, P. C. , Ayasse, M. , & Steiger, S. (2015). Beyond species recognition: Somatic state affects long‐distance sex pheromone communication. Proceedings of the Biological Sciences, 282, 20150832. 10.1098/rspb.2015.0832 PMC4528514 26180067

[ece39699-bib-0047] Costa, J. T. (2006). The Other Insect Societies. Harvard University Press.

[ece39699-bib-0007] Creighton, J. C. , Heflin, N. D. , & Belk, M. C. (2009). Cost of reproduction, resource quality, and terminal investment in a burying beetle. The American Naturalist, 174, 673–684. 10.1086/605963 19775240

[ece39699-bib-0008] Creighton, J. C. , Smith, A. N. , Komendat, A. , & Belk, M. C. (2015). Dynamics of biparental care in a burying beetle: Experimental handicapping results in partner compensation. Behavioral Ecology and Sociobiology, 69, 265–271.

[ece39699-bib-0009] De Gasperin, O. , Duarte, A. , English, S. , Attisano, A. , & Kilner, R. M. (2018). The early‐life environment and individual plasticity in life‐history traits. Ecology and Evolution, 9, 339–351. 30680118 10.1002/ece3.4749 PMC6342119

[ece39699-bib-0010] Eggert, A.‐K. , & Müller, J. K. (1997). Biparental care and social evolution in burying beetles: Lessons from the larder. In A.‐K. Eggert & J. K. Müller (Eds.), The evolution of social behavior in insects and arachnids. Cambridge University Press.

[ece39699-bib-0011] Falk, J. , Wong, J. W. Y. , Kölliker, M. , & Meunier, J. (2014). Sibling cooperation in earwig families provides insights into the early evolution of social life. The American Naturalist, 183, 547–557. 10.1086/675364 24642498

[ece39699-bib-0012] Farchmin, P. A. , Eggert, A.‐K. , Duffield, K. R. , & Sakaluk, S. K. (2020). Dynamic terminal investment in male burying beetles. Animal Behaviour, 163, 1–7.

[ece39699-bib-0013] Filippi‐Tsukamoto, L. , Nomakuchi, S. , Kuki, K. , & Tojo, S. (1995). Adaptiveness of parental care in *Parastrachia japonensis* (Hemiptera: Cydnidae). Annals of the Entomological Society of America, 88, 374–383.

[ece39699-bib-0014] Fox, J. , Weisberg, S. , Adler, D. , Bates, D. , Baud‐Bovy, G. , Ellison, S. , Firth, D. , Friendly, M. , Gorjanc, G. , & Graves, S. (2012). Package ‘car’. Vienna: R Foundation for Statistical Computing:16.

[ece39699-bib-0015] Gray, F. E. , Richardson, J. , Ratz, T. , & Smiseth, P. T. (2018). No evidence for parent–offspring competition in the burying beetle *Nicrophorus vespilloides* . Behavioral Ecology, 29, 1142–1149.

[ece39699-bib-0016] Houston, A. I. , Szekely, T. , & McNamara, J. M. (2005). Conflict between parents over care. Trends in Ecology & Evolution, 20, 33–38. 16701338 10.1016/j.tree.2004.10.008

[ece39699-bib-0017] Javoiš, J. , & Tammaru, T. (2004). Reproductive decisions are sensitive to cues of life expectancy: The case of a moth. Animal Behaviour, 68, 249–255.

[ece39699-bib-0018] Keppner, E. M. , Ayasse, M. , & Steiger, S. (2018). Manipulation of parental nutritional condition reveals competition among family members. Journal of Evolutionary Biology, 31, 822–832. 29573021 10.1111/jeb.13266

[ece39699-bib-0045] Keppner, E. M. , Ayasse, M. , & Steiger, S. (2020). Contribution of males to brood care can compensate for their food consumption from a shared resource. Ecology and Evolution, 10(7), 3535–3543. 10.1002/ece3.6150 32274007 PMC7141021

[ece39699-bib-0019] Kishida, R. , & Suzuki, N. (2010). Effect of carcass size on feeding modes of larvae of *Nicrophorus quadripunctatus* Kraatz (coleoptera: Silphidae). Psyche: A Journal of Entomology, 2010, 1–5.

[ece39699-bib-0020] Kramer, J. , Körner, M. , Diehl, J. M. C. , Scheiner, C. , Yüksel‐Dadak, A. , Christl, T. , Kohlmeier, P. , Meunier, J. , & Boogert, N. (2017). When earwig mothers do not care to share: Parent‐offspring competition and the evolution of family life. Functional Ecology, 9, 40.

[ece39699-bib-0021] Kramer, J. , & Meunier, J. (2019). The other facets of family life and their role in the evolution of animal sociality. Biological Reviews of the Cambridge Philosophical Society, 94, 199–215. 10.1111/brv.12443 29989333

[ece39699-bib-0022] Krams, K. T. , Moore, F. R. , Rantala, M. J. , Mänd, R. , Mierauskas, P. , & Mänd, M. (2015). Resource availability as a proxy for terminal investment in a beetle. Oecologia, 178, 339–345. 25582868 10.1007/s00442-014-3210-5

[ece39699-bib-0023] Kuitunen, M. , Jäntti, A. , Suhonen, J. , & Aho, T. (1996). Food availability and the male's role in parental care in double‐brooded Treecreepers *Certhia familiaris* . Ibis, 138, 638–643.

[ece39699-bib-0024] Lenth, R. (2022). emmeans: Estimated Marginal Means, aka Least‐Squares Means.

[ece39699-bib-0025] Marti, C. D. (1989). Food sharing by sibling common barn‐owls. The Wilson Bulletin, 101, 132–134.

[ece39699-bib-0026] Müller, J.‐K. , Eggert, A.‐K. , & Furlkröger, E. (1990). Clutch size regulation in the burying beetle: *Necrophorus vespilloides* Herbst (coleoptera: Silphidae). Journal of Insect Behavior, 3, 265–270.

[ece39699-bib-0027] Müller, J.‐K. , Eggert, A.‐K. , & Sakaluk, S. K. (1998). Carcass maintenance and biparental brood care in burying beetles: Are males redundant? Ecological Entomology, 23, 195–200.

[ece39699-bib-0028] Pilakouta, N. , Halford, C. , Rácz, R. , & Smiseth, P. T. (2016). Effects of prior contest experience and contest outcome on female reproductive decisions and offspring fitness. The American Naturalist, 188, 319–328. 10.1086/687392 27501089

[ece39699-bib-0029] Pilakouta, N. , Hanlon, E. J. H. , & Smiseth, P. T. (2018). Biparental care is more than the sum of its parts: Experimental evidence for synergistic effects on offspring fitness. Proceedings of the Biological Sciences, 285, 20180875. 10.1098/rspb.2018.0875 PMC6111165 30068674

[ece39699-bib-0030] Pukowski, E. (1933). Ökologische Untersuchungen an Necrophorus. Zeitschrift für Morphologie und Ökologie der Tiere, 27, 518–586.

[ece39699-bib-0031] R Core Team . (2020). R: A language and environment for statistical language and environment for statistical computing. R Foundation for Statistical Computing, Vienna, Austria. https://www.R‐project.org/.4.0.3

[ece39699-bib-0032] Ratz, T. , Kremi, K. , Leissle, L. , Richardson, J. , & Smiseth, P. T. (2021). Access to resources shapes sex differences between caring parents. Frontiers in Ecology and Evolution, 9, 39.

[ece39699-bib-0033] Richardson, J. , Ross, J. , & Smiseth, P. T. (2019). Food deprivation affects egg laying and maternal care but not offspring performance in a beetle. Behavioral Ecology, 30, 1477–1487.

[ece39699-bib-0034] Richardson, J. , Stephens, J. , & Smiseth, P. T. (2020). Increased allocation to reproduction reduces future competitive ability in a burying beetle. The Journal of Animal Ecology, 89, 1918–1926. 32356341 10.1111/1365-2656.13242

[ece39699-bib-0035] Scott, M. P. (1998). The ecology and behavior of burying beetles. Annual Review of Entomology, 43, 595–618. 10.1146/annurev.ento.43.1.595 15012399

[ece39699-bib-0036] Smiseth, P. T. , Dawson, C. , Varley, E. , & Moore, A. J. (2005). How do caring parents respond to mate loss?: Differential response by males and females. Animal Behaviour, 69, 551–559.

[ece39699-bib-0037] Steiger, S. , Richter, K. , Müller, J. K. , & Eggert, A.‐K. (2007). Maternal nutritional condition and genetic differentiation affect brood size and offspring body size in *Nicrophorus* . Zoology (Jena, Germany), 110, 360–368. 17702555 10.1016/j.zool.2007.06.001

[ece39699-bib-0038] Suzuki, S. (2016). When the male determines his provisioning effort: Does the timing of handicapping affect the negotiation between parents in *Nicrophorus quadripunctatus*? Behaviour, 153, 1435–1443.

[ece39699-bib-0039] Tallamy, D. W. , & Wood, T. K. (1986). Convergence patterns in subsocial insects. Annual Review of Entomology, 31, 369–390.

[ece39699-bib-0040] Trumbo, S. T. , & Xhihani, E. (2015). Influences of parental care and food deprivation on regulation of body mass in a burying beetle. Ethology, 121, 985–993.

[ece39699-bib-0041] Williams, G. C. (1966). Natural selection, the costs of reproduction, and a refinement of Lack's principle. The American Naturalist, 100, 687–690.

[ece39699-bib-0042] Wilson, E. O. (1975). Sociobiology: The new synthesis. Harvard University Press.

[ece39699-bib-0046] Wilson, D. S. , & Knollenberg, W. G. (1984). Food discrimination and ovarian development in burying beetles (coleoptera: silphidae: nicrophorus). Annals of the Entomological Society of America, 77(2), 165–170. 10.1093/aesa/77.2.165

[ece39699-bib-0043] Woelber, B. K. , Hall, C. L. , & Howard, D. R. (2018). Environmental cues influence parental brood structure decisions in the burying beetle *Nicrophorus marginatus* . Journal of Ethology, 36, 55–64.

[ece39699-bib-0044] Wong, J. W. Y. , Meunier, J. , & Kölliker, M. (2013). The evolution of parental care in insects: The roles of ecology, life history and the social environment. Ecological Entomology, 38, 123–137.

