# Supplementary material for: Harsh nutritional environment has positive and negative consequences for family living in a burying beetle
Source: Ecol Evol. 2023 Jan 6;13(1):e9699. doi: 10.1002/ece3.9699 (PMC9817192; doi:10.1002/ece3.9699)
Supplement: Supplementary file 1 — Figure S1. Weight of males and females before and after the manipulation of food availability before breeding. Males and females were either well‐fed or food‐deprived. Two connected points show individual beetles’ body mass before and after. Table S1. Carcass consumption during pre‐hatching care: Post hoc test on the interaction of nutrition before breeding and social condition Figure S2. The impact of social condition and carcass size on the decline in carcass mass [g] in the first 48 h after the start of carcass preparation. The left panel shows the decline in carcass mass caused by food‐deprived parents, the right by well‐fed parents. Boxplots show median, interquartile range, food‐deprived parents, the right by well‐fed parents. Boxplots show median, interquartile range, Different letters indicate significant differences (p < .05). Table S2. Clutch size: Post hoc test on the parameter carcass size Figure S3. The impact of social and nutritional condition on clutch size. The left panel shows clutch sizes produced on 2.5 g carcasses, the right on 5 g carcasses. Boxplots show median, interquartile range, minimum/maximum range. Points indicate the original data points. Mean values are indicated by x. Different letters indicate significant differences (p < .05). Table S3‐1. Larval survival rate: post hoc test on carcass size. Figure S4. The impact of social and nutritional condition on larval survival rate. The left panel shows the larval survival rate on 2.5 g carcasses, the right on 5 g carcasses. Boxplots show median, interquartile range, minimum/maximum range. Points indicate the original data points. Mean values are indicated by x. Different letters indicate significant differences (p < .05). Table S3‐2. Larval survival rate: Post hoc test on the interaction of nutrition before breeding and social condition. Figure S4‐2. The impact of nutritional condition and carcass size on larval survival rate. The left panel shows the larval survival rate in biparental brood [file ECE3-13-e9699-s001.pdf]

Supplementary Data – Harsh nutritional environment has positive and negative consequences for family living in a burying beetle.

Figure on beetles' body mass before and after manipulations (1) and Post hoc test results and additional figures with post hoc letters (2)

- Note: Post hoc tests were performed on significant variables using the *emmeans* command of the R package emmeans. Variables were tested together when in a significant interaction, tested separately when no significant interaction with the respective variable was present
- Please be aware of the figure axis and legends - Figures are partly differently arranged than in the main manuscript to be able to include post hoc letters

1. Figure on beetles’ body mass before and after manipulations

Fig S1

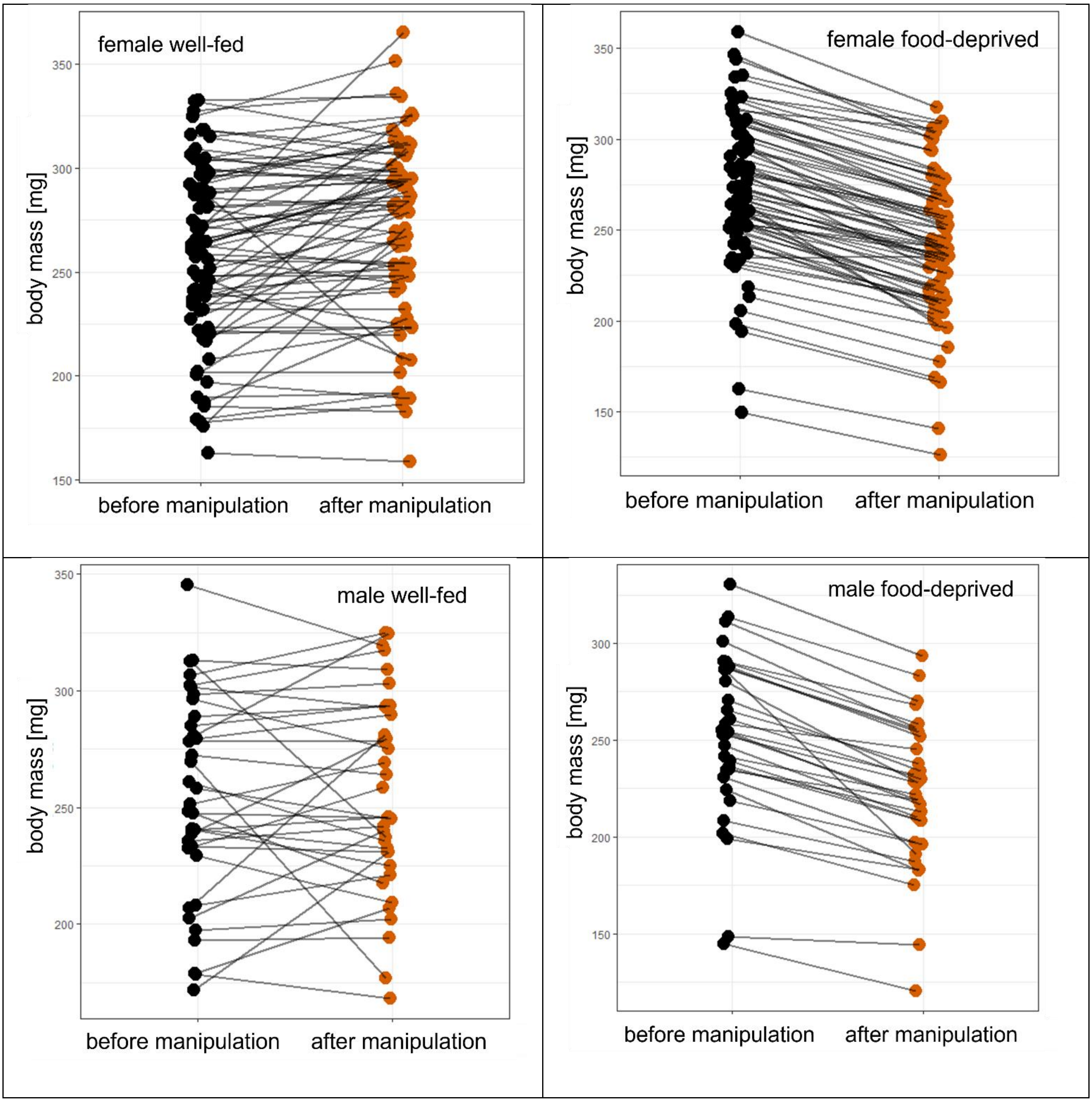

Fig S 1 Weight of males and females before and after the manipulation of food availability before breeding. Males and females were either well-fed or food-deprived. Two connected points show individual beetles` body mass before and after.

2. Post hoc test result and figure with post hoc letters for the response variable carcass consumption

Table S1 Carcass consumption during pre-hatching care: Post hoc test on the interaction of nutrition before breeding and social condition

					estimate	SE	t.ratio	p-value
well-fed	single	-	food-deprived	single	0.0243	0.0441	0.551	0.5823
well-fed	biparental	-	food-deprived	single	0.0809	0.0457	1.772	0.2356
well-fed	biparental	-	well-fed	single	0.0566	0.0462	1.226	0.4445
**food-deprived**	**biparental**	**-**	**food-deprived**	**single**	0.3061	0.0467	6.548	**<.0001**
**food-deprived**	**biparental**	**-**	**well-fed**	**single**	0.2817	0.0472	5.965	**<.0001**
**food-deprived**	**biparental**	**-**	**well-fed**	**biparental**	0.2252	0.0487	4.623	**<.0001**

Fig. S2

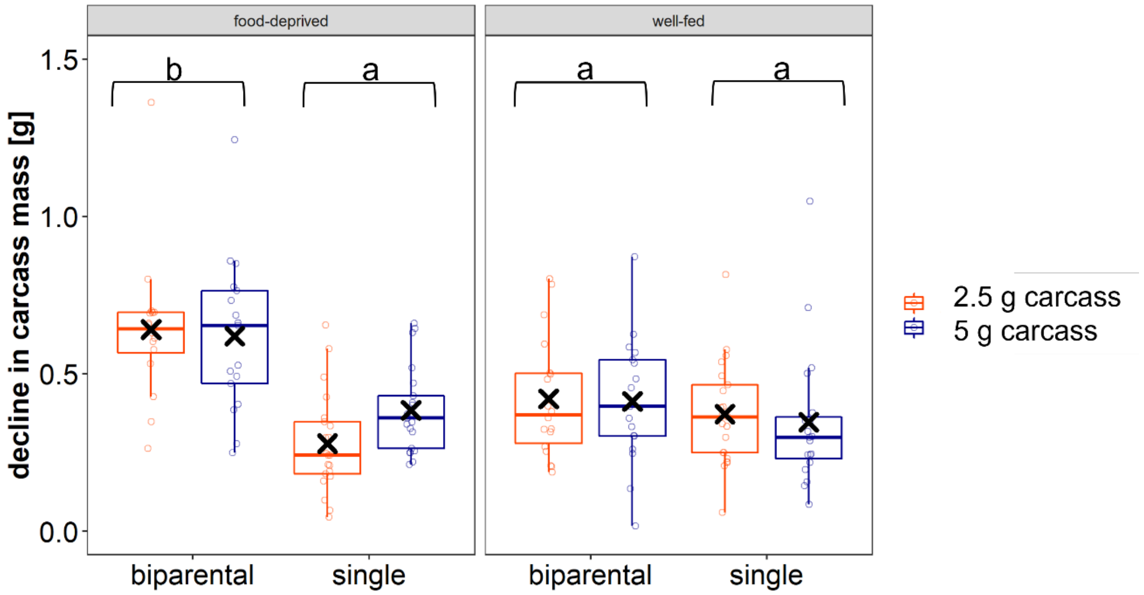

Fig S 2 The impact of social condition and carcass size on the decline in carcass mass [g] in the first 48 h after the start of carcass preparation. The left panel shows the decline in carcass mass caused by food-deprived parents, the right by well-fed parents. Boxplots show median, interquartile range, minimum/maximum range. Points indicate the original data points. Mean values are indicated by x. Different letters indicate significant differences ($p < 0.05$).

2. Post hoc test result and figure with post hoc letters for the response variable clutch size

Table S2 Clutch Size: Post hoc test on the parameter carcass size

			estimate	SE	z.ratio	p-value
**5 g carcass**	-	**2.5 g carcass**	0.394	0.0586	6.728	**<.0001**

Fig. S3

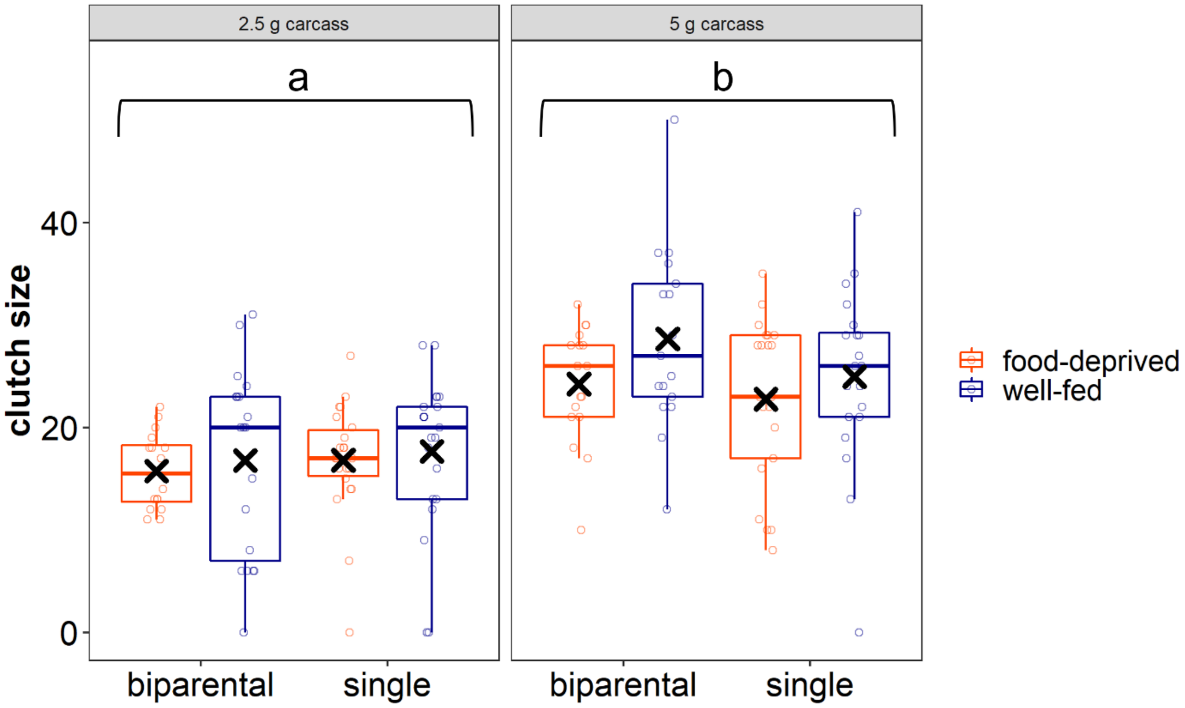

Fig S 3 The impact of social and nutritional condition on clutch size. The left panel shows clutch sizes produced on 2.5 g carcasses, the right on 5 g carcasses. Boxplots show median, interquartile range, minimum/maximum range. Points indicate the original data points. Mean values are indicated by x. Different letters indicate significant differences ($p < 0.05$).

3. Post hoc test result and figure with post hoc letters for the response variable larval survival rate

Table S3-1: Larval survival rate: post hoc test on carcass size

				estimate	SE	df	z.ratio	p.value
	**5 g carcass**	**-**	**2.5 g carcass**	1.07	0.16	Inf	6.817	**<.0001**

Fig. S4-1

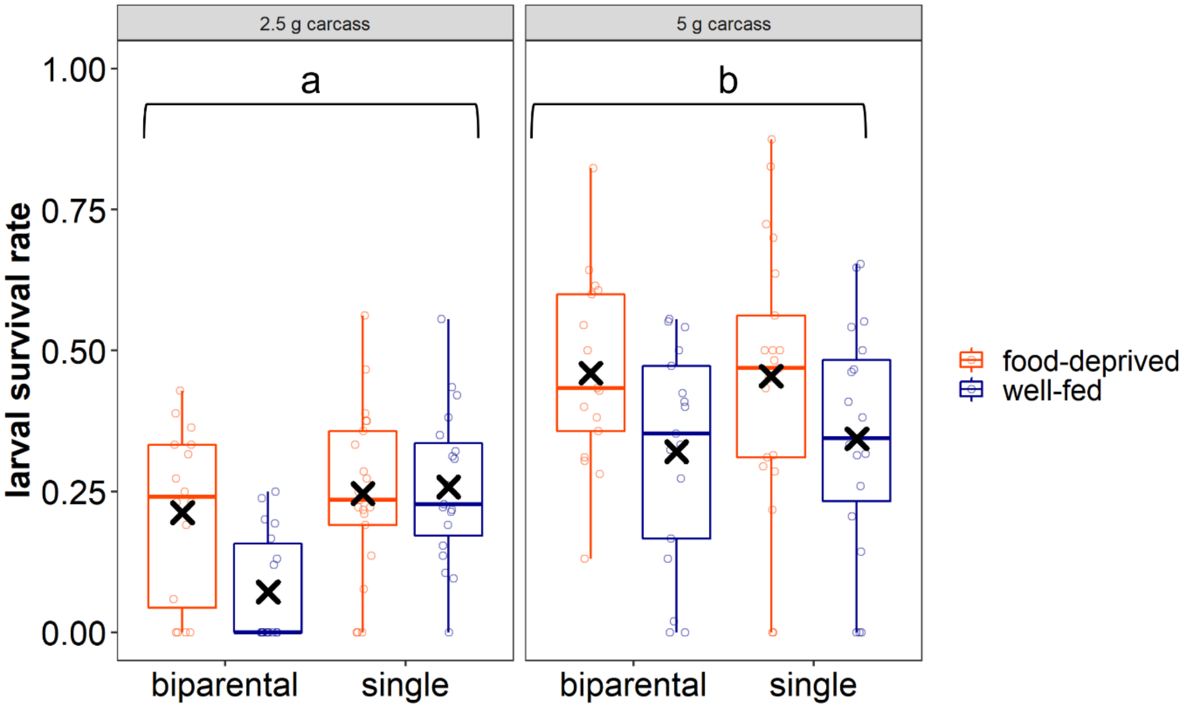

Fig S 4 The impact of social and nutritional condition on larval survival rate. The left panel shows the larval survival rate on 2.5 g carcasses, the right on 5 g carcasses. Boxplots show median, interquartile range, minimum/maximum range. Points indicate the original data points. Mean values are indicated by x. Different letters indicate significant differences ($p < 0.05$).

Table S3-2: Larval survival rate: Post hoc test on the interaction of nutrition before breeding and social condition

						estimate	SE	z.ratio	p.value
	**well-fed**	**single**	**vs**	**well-fed**	biparental	0.8010	0.245	3.272	**0.0043**
	**food-deprived**	**biparental**	**vs**	**well-fed**	biparental	0.9165	0.252	3.630	**0.0014**
	food-deprived	biparental	vs	well-fed	single	0.1155	0.204	0.566	1.0
	**food-deprived**	**single**	**vs**	**well-fed**	biparental	10.023	0.240	4.173	**0.0002**
	food-deprived	single	vs	well-fed	single	0.2013	0.189	1.066	0.8590
	food-deprived	single	vs	food-deprived	biparental	0.0858	0.199	0.432	1.0

Fig. S4-2

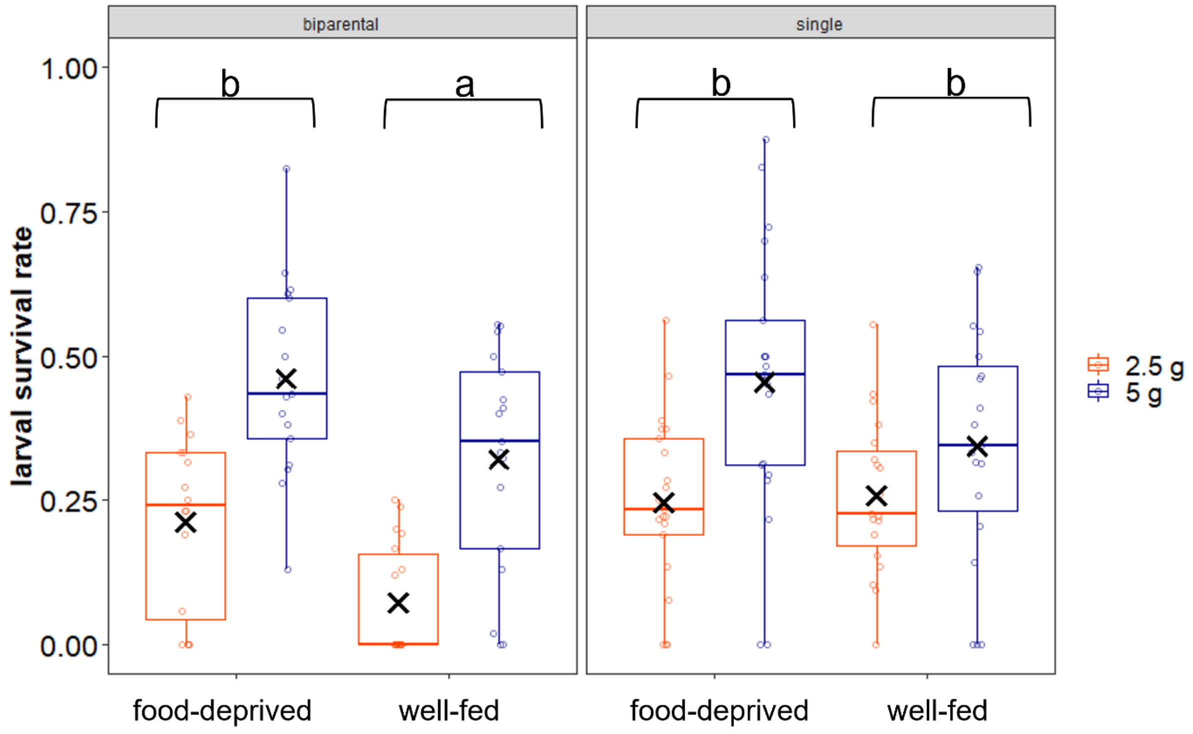

Fig S 4-2 The impact of nutritional condition and carcass size on larval survival rate. The left panel shows the larval survival rate in biparental broods, the right in uniparental broods. Boxplots show median, interquartile range, minimum/maximum range. Points indicate the original data points. Mean values are indicated by x. Different letters indicate significant differences ($p < 0.05$).

4. Post hoc test result and figure with post hoc letters for response variable brood mass

Table S4 Brood mass: Post-hoc tests on the parameters social condition and carcass size

				estimate	SE	t.ratio	p-value
	**single**	**-**	**biparental**	173	59.1	2.927	**0.0041**
	**5 g carcass**	-	**2.5 g carcass**	874	58.5	14.943	**<.0001**

Fig. S5

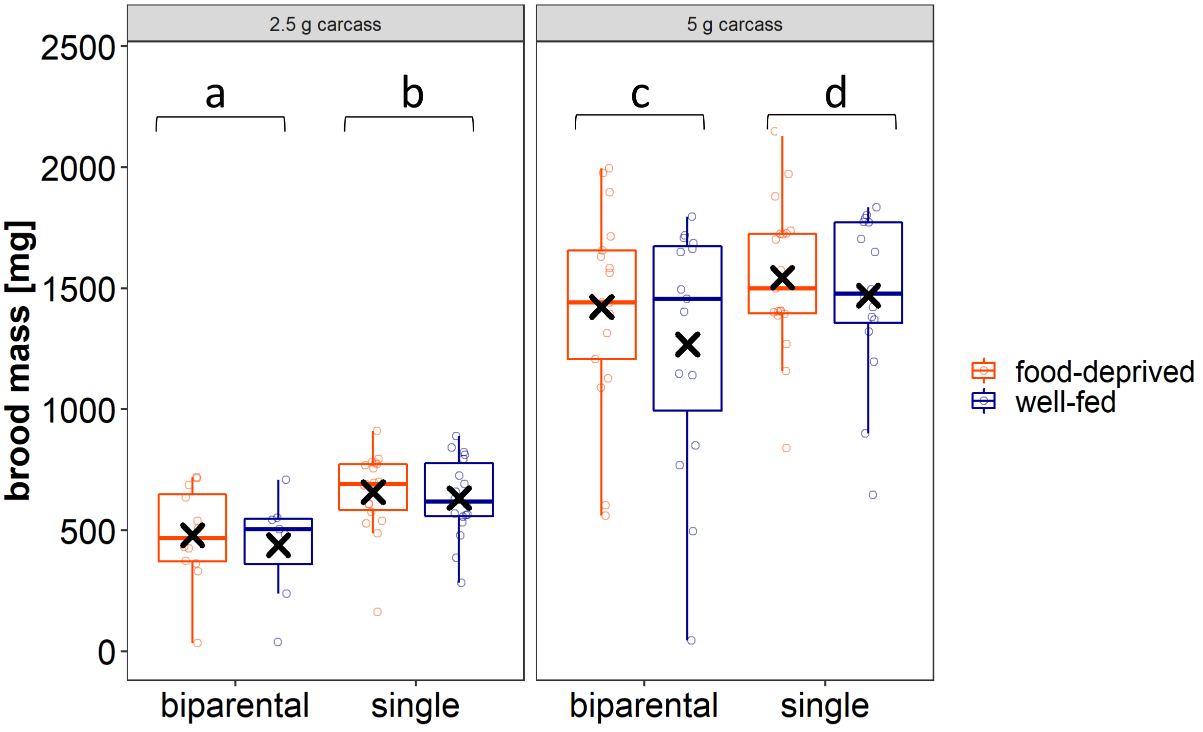

Fig S 5 The impact of social and nutritional condition on total brood mass. The left panel shows the total brood mass produced on 2.5 g carcasses, the right on 5 g. Boxplots show median, interquartile range, minimum/maximum range. Points indicate the original data points. Mean values are indicated by x. Different letters indicate significant differences ($p < 0.05$).
